# Adult onset of ganglioneuroblastoma of the adrenal gland: case report and review of the literature

**DOI:** 10.1186/s40792-015-0062-0

**Published:** 2015-09-11

**Authors:** Elena Bolzacchini, Barbara Martinelli, Graziella Pinotti

**Affiliations:** U.O Oncologia Medica, Azienda ospedaliero-universitaria Ospedale di Circolo e Fondazione Macchi, Viale Borri, 57, 21100 Varese, Italy

**Keywords:** Adrenal, Ganglioneuroblastoma, Adult onset

## Abstract

Ganglioneuroblastoma (GBN) is a malignant neoplasm of the autonomic nervous system. Adult onset of ganglioneuroblastoma is extremely rare. Only 16 cases have been reported in English literature, to date. Surgery represents the first-line therapy for the treatment of ganglioneuroblastoma. Radiation therapy is indicated in patients with localized unresectable disease. Chemotherapy is reserved for metastatic disease.

We present the case of a 63-year-old man affected by ganglioneuroblastoma of the adrenal gland. The diagnosis was made incidentally. The tumor, measuring 5 × 3 cm, was successfully surgically removed.

## Background

Ganglioneuroblastoma (GBN) is a malignant neoplasm of the autonomic nervous system; it originates from primitive neuroectodermal cells of the neural crest that migrate during embryonic life giving rise to the sympathetic ganglia and the adrenal medulla. Ganglioneuroblastoma represents a subgroup of neuroblastoma tumors with a prominent, mature ganglion cell differentiation, usually located in the adrenal gland (the most frequent site) but also in the posterior mediastinum, the retroperitoneum, and the brain [1]. Adult onset is extremely rare. Only 16 cases of adrenal ganglioneuroblastoma of the adult are reported in English literature.

## Case presentation

**Fig. 1:**
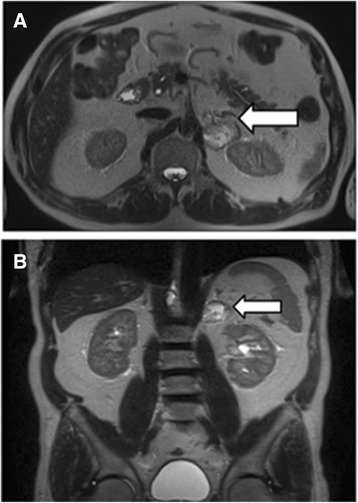
Abdominal MRI scan. **a** Coronal MRI scan of the abdomen. **b** Axial MRI scan of the abdomen. The *arrows* indicate the surrenal mass: the signal was highly inhomogeneous and the central area was partially fluid

In November 2013, a 63-year-old Caucasian man underwent a CT scan for recurrent renal stones. A mass of the left adrenal gland measuring 5 × 3 cm was incidentally found. The mass was irregular and hypodense and had inhomogeneous contrast uptake, especially in the arterial phase. Magnetic resonance imaging (MRI) confirmed the mass: the signal was highly inhomogeneous and the central area was partially fluid (see Fig. 1a, b). The radiologist’s report excluded the diagnosis of adenoma but could not define the lesion.

The patient was in good clinical condition and asymptomatic. The complete physical examination was negative. Blood pressure and pulse were normal. Results of the complete blood count, plasma levels of electrolytes, tests of coagulation, kidney and liver function, and urinalysis were normal. For the history of nephrolithiasis, phosphocalcic profile was analyzed and primary hyperparathyroidism as a cause of kidney stones was excluded. Testing for adrenal gland function were all normal: in particular, urinary metanephrines and urinary free cortisol were normal, DHEAS = 1.7 mcg (normal range: 0.2–3.3), 17-OH-Pg = 1.1 ng/ml (normal range: 0.4–3.3), aldosterone = 291 pg/ml (normal range: 70–300 pg/ml), renin = 13.2 MCU/ml (normal range: 7–76), aldosterone-renin ratio = 2.2 (normal range <3.4). All the tests were made in order to rule out the possibility of a functioning lesion. In particular, blood tests excluded hypercortisolism, so the patient could undergo surgery without risks.

A radical adrenalectomy with complete excision of the lesion was performed. The surgery was radical and without postoperative complication.

**Fig. 2:**
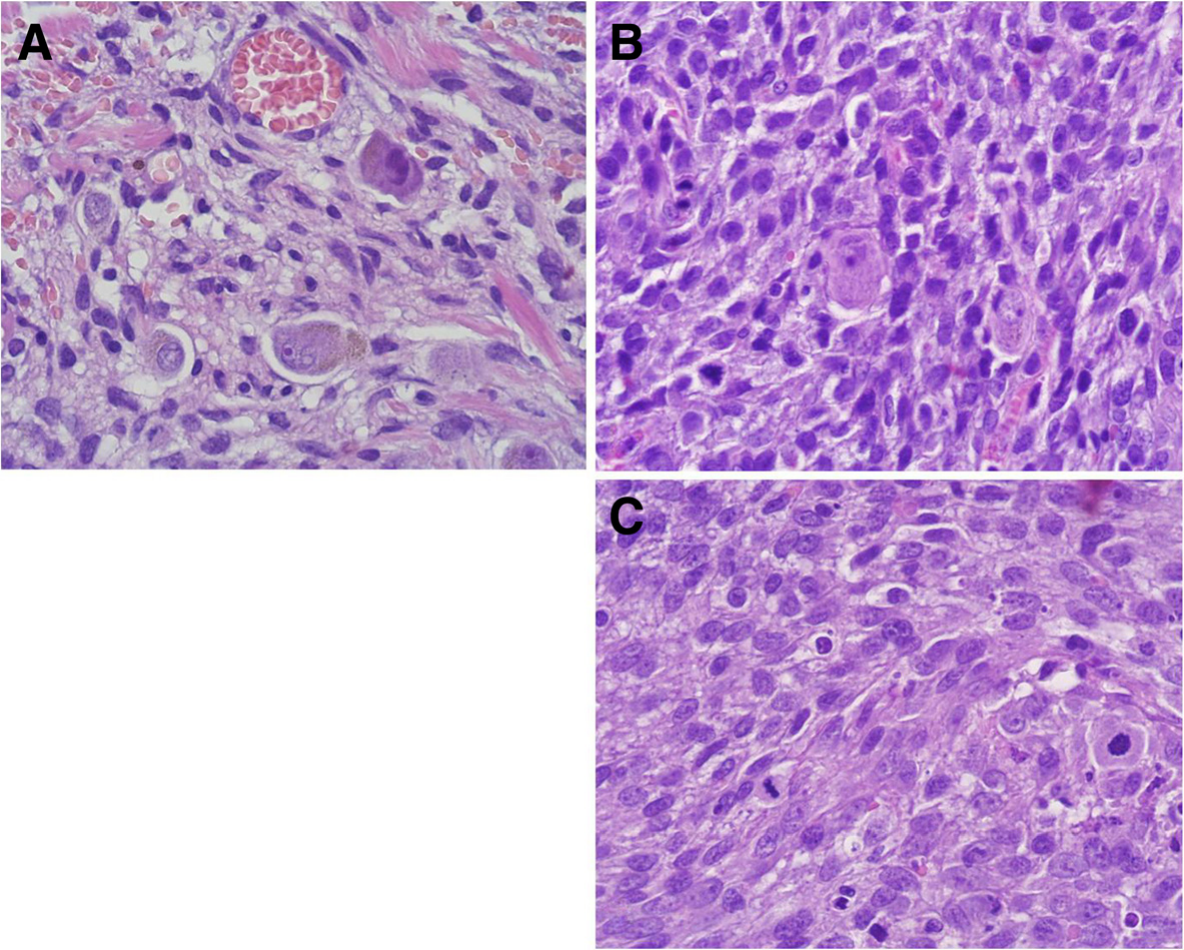
The histopathological examination of the tumor showed a proliferation of atypical ganglion cells, in many of which neuro-melaninic pigment was evident, interspersed in a fibrillary matrix (**a**). A nodular proliferation of poorly differentiated spindle cells (**b**) with a high mitotic index (27 mitosis per HPF, ×400) (**c**) was also observed

The surgical sample sent for pathological examination was composed of the adrenal gland and of a nodular mass of 4.5 cm in its greater diameter, with a hemorrhagic and partially necrotic cut surface. The histopathological report described an encapsulated proliferation, which consisted of atypical ganglion cells, in many of which neuro-melaninic pigment was evident, interspersed in a fibrillary matrix (Fig. 2a). In addition, a nodular proliferation of poorly differentiated spindle cells with a high mitotic index (27 mitosis per HPF, ×400) and areas of necrosis was observed (Fig. 2b, c). The neoplastic cells were partially immunoreactive for synaptophysin and neurofilament, while they were negative for S100, Melanin A, CD99, CD31, CD34, podoplanin, cytokeratin AE1/AE3, alpha-inhibin, and actin. These findings were consistent with a nodular ganglioneuroblastoma.

As guidelines suggest, the patient underwent total body CT scan and mIBG scintigraphy after the surgery and both were negative. The patients will continue follow-up schedule every 3 months.

### Discussion

Neuroblastic tumors arise from sympathetic nervous system and are divided into three major categories according to Shimada et al*.* [2]: neuroblastoma, ganglioneuroblastoma, and ganglioneuroma. Ganglioneuroblastoma is a composite tumor containing both primitive neuroblasts and mature ganglion cells. It is a malignant tumor but less aggressive than neuroblastoma.

The International Neuroblastoma Pathology Classification (INPC), established in 1999 and revised in 2003, re-defined the histological features and proposed 4 tumor categories: NB (neuroblastoma), GNB-intermixed, GN (ganglioneuroma), GNB-nodular (classical). The four categories are divided in two distinct prognostic groups: Favorable Histology (FH) and Unfavorable Histology (UH) [3, 4] (see Table 1).

**Table 1:** International Neuroblastoma Pathology Classification

Category and subtype	Pathology classification
Neuroblastoma (Schwannian stroma-poor)	FH and UH subgroups, based on the combination of age, grade of neuroblastic differentiation, and MKI
Undifferentiated	
Poorly differentiated	
Differentiating	
Ganglioneuroblastoma, intermixed (Schwannian stroma-rich)	FH
Ganglioneuroma(Schwannian stroma-dominant) FH	FH
Maturing	
Mature	
Ganglioneuroblastoma, nodular (Schwannian stroma-rich/stroma-dominant and stroma-poor)	UH^a^

*FH* favorable histology, *UH* unfavorable histology, *MKI* mitosis-karyorrhexis index

^a^Tumors in this category were classified into an unfavorable histology group according to the International Neuroblastoma Pathology Classification

The tumor type of the current case was GBN-nodular (classical), unfavorable histology.

The International Classification INSS (revised in 1993) is currently used for the staging of the disease (see Table 2) [5]. Important prognostic factors are age at the diagnosis (children <1 year old have the most favorable prognosis), primary site of the tumor (retroperitoneum and adrenal gland tumors bear a worse prognosis than mediastinum lesions), histology and stage of disease according to INSS [6].

**Table 2:** The International Neuroblastoma Staging System (INSS)

Stage/Prognostic group	Description
Stage 1	Localized tumor with complete gross excision, with or without microscopic residual disease; representative ipsilateral lymph nodes negative for tumor microscopically (i.e., nodes attached to and removed with the primary tumor may be positive).
Stage 2A	Localized tumor with incomplete gross excision; representative ipsilateral non-adherent lymph nodes negative for tumor microscopically.
Stage 2B	Localized tumor with or without complete gross excision, with ipsilateral non-adherent lymph nodes positive for tumor. Enlarged contralateral lymph nodes must be negative microscopically
Stage 3	Unresectable unilateral tumor infiltrating across the midline, with or without regional lymph node involvement; or localized unilateral tumor with contralateral regional lymph node involvement; or midline tumor with bilateral extension by infiltration (unresectable) or by lymph node involvement. The midline is defined as the vertebral column. Tumors originating on one side and crossing the midline must infiltrate to or beyond the opposite side of the vertebral column.
Stage 4	Any primary tumor with dissemination to distant lymph nodes, bone, bone marrow, liver, skin, and/or other organs, except as defined for stage 4S.
Stage 4S	Localized primary tumor, as defined for stage 1, 2A, or 2B, with dissemination limited to skin, liver, and/or bone marrow (by definition limited to infants younger than 12 months). Marrow involvement should be minimal (i.e., <10 % of total nucleated cells identified as malignant by bone biopsy or by bone marrow aspirate). More extensive bone marrow involvement would be considered stage 4 disease. The results of the mIBG scan, if performed, should be negative for disease in the bone marrow.

*mIBG* metaiodobenzylguanidine

Moreover, it is important to consider biological factors, in particular amplification of N-MYC (which is amplified in 20–30 % of neuroblastomas) and deletion of the short arm of chromosome 1 that are related to poor prognosis [7, 8].

Ganglioneuroblastoma is a pediatric tumor and adult onset is extremely rare. In the literature, less than 50 cases of adult patients are reported [9]; the most frequent site is the adrenal gland; other sites are the retroperitoneum, the brain, and the mediastinum. In the adrenal gland, only 17 cases of GNB of the adult are reported, including our patient (see Table 3) [9–23]. Most of them (14/17) were male, from 20 to 67 years old. Six patients (35 %) showed distant metastasis or local recurrence; metastatic sites were bone or bone marrow in three cases (17 %), lymph node in two cases (11 %), and liver in two cases (11 %). All patients underwent surgery and two of them underwent also radiotherapy and chemotherapy.

**Table 3:** Results

First author (year)	Age(years)	Sex	Size(cm)	Metastasis	Treatment	Survival
Butz (1940) [10]	25	M	(−)	Liver	(−)	(−)
Cameron (1967) [11]	58	F	(−)	None	Surgery	3.5 years alive
Takahashi (1988) [12]	21	M	8.8	Lymph node	Surg rad, chemo	8 months
Koizumi (1992) [13]	47	F	9	Bone marrow	(−)	3 months
Kishikawa (1992) [14]	29	M	11	Bone	Surg	(−)
Higuchi (1993) [15]	29	M	11	Bone	Surg	10 months, alive
Hiroshige (1995) [9]	35	M	10	None	Surg	24 months
Mehta (1997) [16]	22	M	9	(−)	Surg	(−)
Rousseau (1998) [17]	(−)	F	(−)	Liver	Surg rad, chemo	(−)
Fujiwara (2000) [18]	25	M	(−)	None	Surg	5 years
Leavitt (2000) [19]	67	M	(−)	None	Surg	(−)
Slapa (2002) [20]	20	F	18	None	Surg	12 months alive
Koike (2003) [14]	50	M	4.5	(−)	Surg	30 months
Gunlusoy (2004) [21]	59	M	17	Lymph node	Surg	(−)
Mizuno (2006) [22]	53	M	11	Spine	Surg	30 months, alive
Gupta (2007) [23]	40	M	(−)	None	Surg	(−)
Present case (2014)	63	M	5	None	Surg	6 months alive

(−) not available

Treatment guidelines were derived from pediatric experience. The therapeutic strategy includes surgery, radiotherapy, and chemotherapy. Patients with N-MYC amplification or deletion of the short arm of chromosome 1 have aggressive disease, regardless of age and stage of disease. If the tumor is considered radically resectable, surgery represents the treatment of choice. Because of the high possibility of infiltration of regional lymph nodes, local lymph node dissection is recommended [24, 25].

If the tumor is considered unresectable (stage 3), a diagnostic biopsy should be performed and treatment options are radiotherapy or cytoreductive chemotherapy. Chemotherapy is the treatment of choice in metastatic disease (stage 4) [26].

Active drugs in pediatric population are cyclophosphamide vincristine, adriamycin, and combinations with platinum and etoposide [27]. In high-risk patients (stage 4 or any stage with N-MYC amplification), chemotherapy schedules with higher dose intensity are recommended. Topotecan [28] and temozolomide [29] have recently demonstrated a significant antitumor activity in patients with relapsed disease. In stage 4, the role of radiation therapy is controversial.

Recurrence of disease occurs mostly in the first 2 years after surgery. Attention to the symptoms and careful physical examination are fundamental. The patient should be examined every 3 months in the first and second year, then every 6 months. Complete blood count, urinary catecholamine analysis, and imaging of the site of the primary tumor (ultrasound or RX scan) should be performed at every examination.

It is recommended to perform MIBG scintigraphy every 6 months during the first and second year [1].

Few data are available regarding treatment of adults affected by GBN. In fact, it is not clear if chemotherapy schedules of pediatric studies are suitable and effective for metastatic disease in adults. Prognosis of GBN of the adult also remains uncertain because of the poorness of long-term data.

## Conclusions

Ganglioneuroblastoma is typically a pediatric disease. Few cases of GBN of the adult are described from the literature. Surgery is the treatment of choice in stage 1 and 2 (children and adult), radiotherapy as a role in stage 3 disease, and chemotherapy is reserved for metastatic disease. Few data are available regarding the efficacy of chemotherapy in stage 4 in the adults. Because of the rarity of the pathology, all cases should be included in the rare cancer network.

## Consent

Written informed consent was obtained from the patient for publication of this case report and any accompanying images. A copy of the written consent is available for review by the Editor-in-Chief of this journal.

## Abbreviations

GBN: ganglioneuroblastoma
